# Upper limb kinematics during the first year after stroke: the stroke arm longitudinal study at the University of Gothenburg (SALGOT)

**DOI:** 10.1186/s12984-020-00705-2

**Published:** 2020-06-15

**Authors:** Gyrd Thrane, Katharina Stibrant Sunnerhagen, Margit Alt Murphy

**Affiliations:** 1 Department of Health and Care Sciences, UiT The Arctic University of Norway, Postboks 6050 Langnes, 9037 Tromsø, Norway; 2 Institute of Neuroscience and Physiology, Department of Clinical Neuroscience, Rehabilitation Medicine, Sahlgrenska Academy, University of Gothenburg, Gothenburg, Sweden

**Keywords:** Stroke, Upper extremity, Kinematics, Movement analysis, Recovery, Motor impairment

## Abstract

**Background:**

Reduction of compensation and improved movement quality indicate recovery after stroke. Since clinical measures alone are often inadequate to distinguish between behavioral recovery and compensation, kinematic analysis of functional tasks has been recommended.

**Objective:**

To quantify longitudinal changes and residual deficits in movement performance and quality during the first year after stroke using kinematic analysis of drinking task.

**Methods:**

A total of 56 participants with first ever stroke causing upper extremity impairment were extracted from a non-selected stroke unit cohort (Stroke Arm Longitudinal Study at the University of Gothenburg-SALGOT). Participants needed to able to perform the drinking task with the more-affected arm at least on 2 occasions out of 6 (3 days, 10 days, 4 weeks, and 3, 6, and 12 months) during the first year to be included. A cohort of 60 healthy individuals was used as reference. Longitudinal changes were analyzed using linear mixed models.

**Results:**

Movement time, number of movement units, peak angular velocity of the elbow, peak hand velocity, and trunk displacement improved significantly over the first 3 months with a peak at 6 months. Movement time and peak hand velocity reached levels comparable to healthy at 3 months, but number of movement units, peak elbow angular velocity, trunk displacement, and arm abduction remained different from healthy over the first year after stroke.

**Conclusions:**

Even when the recovery patterns of kinematics follow the known nonlinear pattern, not all kinematic measures reach the levels in par with healthy controls at one year post stroke. Since the number of movement units, peak angular velocity, trunk displacement, and arm abduction remained impaired over the first year, they might be the most suited measures to distinguish behavioral recovery from compensation strategies.

**Trial registration:**

ClinicalTrials: NCT01115348. 4 May 2010. Retrospectively registered.

## Introduction

A stroke can significantly impact a person’s ability to participate in many activities of daily living. Patients with stroke regain most of their independence to perform basic daily activities within the first 60 days after stroke [1]. However, stroke patients can still experience difficulties with basic hand activities during the late subacute and chronic phases after stroke, and these difficulties may persist for years [2–4].

Proper motor function is an important prerequisite for the effective use of upper extremities during different activities. Recovery of motor impairment can be characterized by the reappearance of premorbid movement patterns during task accomplishment [5]. A reduction of synergic and compensatory motor patterns resulting in improved quality of movement may be an indicator of functional restoration facilitated by neural plasticity [5, 6]. Upper-extremity activities may also improve as a result of new motor patterns arising from an adaptation of the remaining motor elements or compensation by other body segments [5]. Sensorimotor impairments in the upper limbs can be measured by assessment tools such as the Fugl-Meyer Assessment of Upper Extremity (FMA-UE) [7, 8]. In order to capture improvements in upper extremity motor function during the first 3 months post stroke, measures of impairment are of particular interest. Although, to detect improvements in motor function at later phase post stroke, more sensitive outcome measures might be needed [9–12].

Kinematic analysis using optoelectronic systems provide precise and detailed measurements of movement performance and quality. A resent task force for stroke recovery metrics has recommended both 2D planar reaching and 3D functional reach-to-grasp task for measuring behavioral restitution of the upper limb [13]. The measurements in 3D using functional tasks are of particular interest since they provide quantifiable metrics on the quality of movement during a natural task that is well known to the performer [14, 15]. Seven kinematic variables, movement time, number of movement units, peak angular elbow velocity, peak hand velocity, relative time to peak hand velocity, arm abduction and trunk displacement, are known to capture most of the overall variation in kinematic performance after stroke [14].

Previous research can also guide selection of kinematic metrics to capture recovery after stroke. A previous longitudinal study reported that 3 kinematic variables, movement time, smoothness, and hand opening during a reach-to-grasp task, improved up to 8 weeks after stroke [16]. Another longitudinal study from van Dokkum et al. [17] reported that the number of velocity peaks during a reach-to-grasp task together with the time after stroke explained 62.5% of the variation in FMA-UE over a 3-month rehabilitation period. Movement time, smoothness, and trunk displacement during a drinking task are sensitive to capture clinically relevant improvements in upper-extremity activity capacity, defined as 6 or more points change in the Action Research Arm test, during the first 3 months after stroke [18]. To advance the research further, analysis with repeated measurements of kinematic variables, obtained during the potential window of recovery, is needed to verify which metrics are useful markers that indicate improvement in movement quality and recovery.

The aim of this study was to quantify longitudinal development in movement performance and quality during the first year after stroke using kinematic analysis of a drinking task and identity which metrics that reach comparable level with healthy controls.

## Methods

### Study design

The participants in this study were included from the Stroke Arm Longitudinal Study at the University of Gothenburg (SALGOT). All participants were patients with first-time stroke who were admitted to the stroke unit at the Sahlgrenska University Hospital during an 18-month period in 2009–2010. The inclusion criteria were the following: Age > 18 years, admission to the stroke unit within 3 days of stroke onset, and having upper-extremity motor impairment at 3 days defined as an Action Research Arm Test score below the maximum (57 points). The exclusion criteria were the following: not communicating in Swedish prior to the stroke, living outside the Gothenburg urban area (> 35 km from Sahlgrenska Hospital), prior upper-extremity injury or condition that limited functional use of the affected arm and hand, and severe multi-impairment or diminished physical condition before the stroke that could affect arm function. To participate in the kinematic measurement, the patient was required to have the ability to use their more-affected arm to drink from a glass. The included patients were assessed at 3 days, 10 days, 4 weeks, and 3, 6, and 12 months after stroke. We included the patients enrolled in the SALGOT cohort who had kinematic measurements performed at a minimum of 2 timepoints. In addition, 60 healthy individuals with commensurable age and sex distribution who did not present any medical conditions affecting their arm functions were recruited as a reference for the kinematic testing. The kinematic data from the SALGOT cohort have previously been included in a previous cross-sectional study investigating residual impairments in high functioning stroke (FMA-UE > 60 points) [19], in a study investigating changes in kinematics in high functioning stroke between 3 and 12 months post stroke [12], and in a study investigating responsiveness in three kinematic variables between two timepoints in the subacute phase after stroke [18]. In contrast to these previous studies, the current study quantifies the longitudinal change in 7 kinematic variables for the entire available cohort over all 6 time points included in the SALGOT protocol from 3 days to 12 months after stroke and evaluates the residual impairments in comparisons to healthy subjects.

### Kinematic analysis

Kinematic measurements were obtained during a standardized drinking task [14, 20]. The participants were instructed to sit with their back against the back of the chair during the completion of the task, but were not constrained in that position. A hard-plastic glass, 7 cm in diameter and 9.5 cm height, was placed on a table in the body midline, 30 cm from the edge of the table. The chair and table height were adjusted so that the participant had a 90° angle in their knee and hip joints. The upper arm was adducted close to the body and the forearm horizontal with 90° angle in the elbow joint. The pronated hand rested on the table with the wrist line close to the edge of the table. In this position the glass could be reached with the less-affected arm without trunk forward displacement. The task performed included 5 phases: reaching and grasping the glass, lifting the glass from the table and bringing it to the mouth, taking one sip of water, placing the glass back down behind a line marked on the table, and returning the arm to its initial position. A motion capture system (ProReflex MCU240 Hz, Qualisys, Gothenburg, Sweden) with 5 optoelectronic cameras was used to obtain the movement data. Retroreflective markers were placed on the third metacarpophalangeal joint of the hand, styloid process of the ulna on the wrist, lateral epicondyle of the elbow, middle part of the acromion on the right and left shoulders, upper part of the sternum, forehead, and upper and lower edges of the glass. The task was performed 5 times at a comfortable speed, with the mean time of the 3 middle trials used for the analysis [14]. We used data from the more-affected arm of the patients recovering from stroke and the non-dominant hand of the healthy participants.

A previous study using principal component analysis showed that kinematic performance of drinking task can mainly be explained by two major factors including five components [14]. The components comprised temporal (e.g. movement time, velocity) and spatial measures (e.g. joint angles and trunk displacement). In the current study, 7 kinematic variables that have shown to be reliable, valid and responsive in stroke population, covering both temporal and spatial aspects of movement performance, were included in the analysis [14]. The tangential velocity of the 3rd metacarpal marker was used to define the start and endpoint of each movement phase and to calculate the following variables: number of movement units (n), peak velocity during reaching (cm/s), and relative time to peak velocity (%). The total movement time (s) was the time required to complete all 5 phases of the drinking task and was measured from the point where the hand marker surpassed 2% of the peak velocity of the reaching phase to the point where it returned back to 2% of the peak velocity of the returning phase. The number of movement units was calculated as the number of velocity peaks during the task, excluding the drinking phase [15]. A difference between the local minimum and the next maximum velocity value that exceeded the 20 mm/s amplitude limit signified a velocity peak. The time between 2 subsequent peaks had to be a minimum of 150 ms to be defined as a movement unit [14]. A smooth movement has a bell shape profile with 1 predominant velocity peak. Multiple peaks in velocity profile signify repetitive acceleration and deceleration indicating an unsmooth and less efficient movement. The minimum number of movement units was 4, which was one for each movement phase. The peak hand velocity was defined as maximal tangential velocity during the reaching phase. The relative time to peak velocity was calculated as percentage of time to peak hand velocity during the reaching phase.

The elbow angle was calculated by the vectors that joined markers at the wrist and elbow, and at the elbow and shoulder. The joint angle at maximal extension and the peak angular velocity of the elbow joint during reaching were computed. Trunk displacement was defined as the maximal forward displacement (mm) of the sternal marker from its initial position during the entire task. The maximal angle between the vector that joined the shoulder and elbow marker projected to the frontal plan and the vertical vector from the shoulder marker was used as an approximation for the shoulder abduction angle during the drinking phase [14, 20].

### Clinical scales

We gathered information from the stroke group, including the stroke type (hemorrhagic or ischemic) and the time elapsed since the stroke. Stroke severity was assessed using the National Institutes of Health Stroke Scale (NIHSS) score at the time of hospital admission [21, 22]. NIHSS scores range from 0 to 42, with a score < 6 indicating a mild stroke and a score > 20 indicating a severe stroke. Motor impairment of the more-affected arms was assessed using the FMA-UE. The FMA-UE includes 33 items that assess movement, coordination, and reflex actions of the shoulder, elbow, forearm, wrist, and hand. Each item is scored on a 3-point ordinal scale (0, cannot perform; 1, performs partially; and 2, performs fully); the test has a total possible score range of 0–66 [8]. The sensations for light touch and position were assessed using the FMA domain for sensation (FMA-Sensation), which has a total score range of 0–12; a score of 12 indicates normal sensation [8]. At each visit, the patients with stroke reported the number of treatment sessions they had with an occupational or physical therapist.

### Statistical analyses

The statistical analyses were performed using R language for statistical computing, version 3.6.2 [23]. The distribution of the included variables was evaluated by visually inspecting histograms and qq-plots.

As some of the kinematic variables, movement time, number of movement units and trunk displacement, had a non-normal and skewed distribution, the obtained kinematic variables were described with medians and quartiles. The comparison between healthy controls and stroke patients were done with a Mann-Whitney U test. For each kinematic variable, the *p*-values of the 6 repeated tests were adjusted using the Holms method [24] and presented in the results.

For longitudinal analysis, all kinematic variables were converted into a percentage of normal performance. A normal performance was defined as the median value for the healthy participants. Inverse values were used for the following variables in which larger values indicated a worse outcome: Movement time, number of movement units, arm abduction, and trunk displacement. Percentage score for inversely transformed variables were:

$$ Percentage\ of\ normal= 100\ast \left( 1/ Obtained\ value\right)/\left( 1/ Median\ value\ in\ healthy\right) $$

For the rest of the variables, variables where a higher obtained score indicate better outcome the percentage score were calculated with the following formula:

$$ Percentage\ of\ normal= 100\ast Obtained\ value/ Median\ value\ in\ healthy $$

A value of 100 reflected the median value for healthy participants in every scale, which made them easier to compare. Thus, for all percentage scores, a higher value indicates a better performance. Outliers, more than 3 SDs from the mean, were noted for the relative time to peak hand velocity (1 out of 243), peak elbow angular velocity (2 out of 243), arm abduction during drinking (3 out of 243) and trunk displacement (1 out of 243). Outliers were evaluated for possible measurement errors, but all variables were considered novel observations and kept in the analyses.

Longitudinal changes were assessed using linear mixed models in the Lme4 package [25]. For each converted (percentage of normal) kinematic variable, a model that included both the fixed effect of intercept and time, and the random effect of the intercept was evaluated. Timepoint was included as a categorical variable. The timepoint with the best mean performance was set as the reference point. The *p*-values were adjusted for multiple comparisons with a simultaneous interference procedure [26]. While the estimates from linear mixed models are robust to missing values, extra additional subgroup analysis of patients that were included at 4 weeks or later after stroke were performed to ensure that the late inclusion did not had an impact on the results.

## Results

**Fig. 1:**
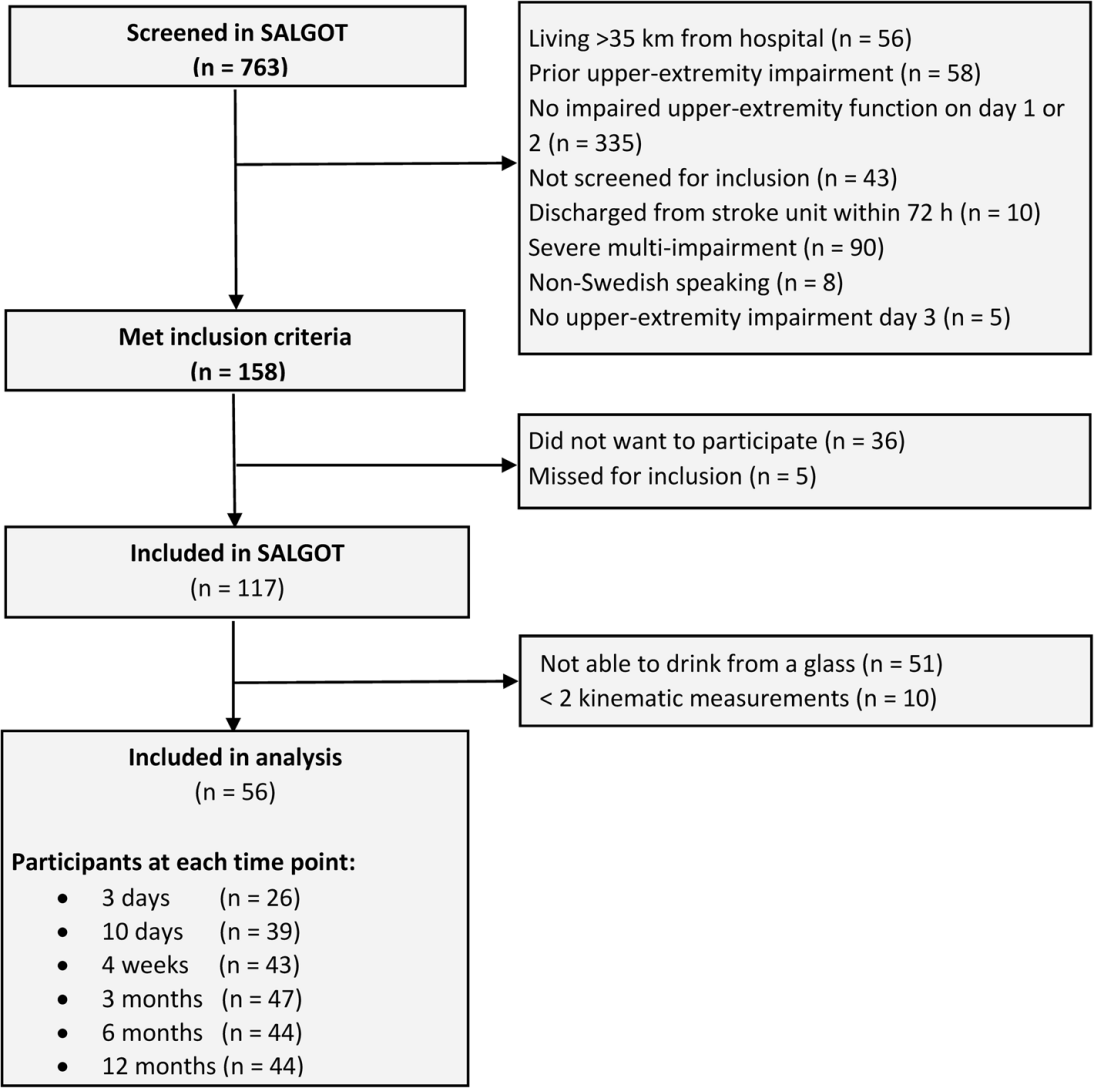
Study inclusion process and the samples taken for analysis. SALGOT, Stroke Arm Longitudinal Study at the University of Gothenburg; FMA, Fugl-Meyer Assessment

A total of 763 patients admitted to stroke unit were screened, 117 were met the inclusion criteria of the SALGOT, and 56 of those who were able to execute the drinking task at least in two occasions during the first year post stroke were included in the current study (Fig. 1). Out of 56 included patients, about 73% (*n* = 41) were included within 10 days post stroke. Six and four patients entered the study at 4 weeks and 3 months, respectively, due to insufficient motor function to perform the drinking task with the affected arm at earlier time points. The mean age of the study group was 64 years (SD 13.4) and a larger proportion was men (62.5%). Stroke severity at admission measured by the NIHSS score ranged from 0 to 24 with a median of 4 points, which indicates that most patients had moderate and mild stroke severity. Table 1 describes the characteristics of the included patients at each timepoint. The healthy reference group included 27 females (45%) and 33 males (55%); the mean age was 63.4 years (SD 12.6).

**Table 1:** Characteristics of patients with stroke included in analysis at each time point

	Overall	3 days	10 days	4 weeks	3 months	6 months	12 months
**N**	56	26	39	43	47	44	44
**Age, admission, mean (SD)**	64.0 (13.4)	64.0 (13.2)	61.9 (13.2)	63.7 (11.9)	62.9 (12.3)	63.4 (13.0)	64.4 (12.3)
**Sex, female, n (%)**	21 (37.5)	7 (26.9)	12 (30.8)	18 (41.9)	18 (38.3)	18 (40.9)	19 (43.2)
**Stroke Location, n (%)**							
**Right**	30 (53.6)	17 (65.4)	20 (51.3)	23 (53.5)	26 (55.3)	25 (56.8)	23 (52.3)
**Left**	22 (39.3)	7 (26.9)	16 (41.0)	18 (41.9)	19 (40.4)	17 (38.6)	18 (40.9)
**Bilateral**	3 (5.4)	2 (7.7)	2 (5.1)	1 (2.3)	1 (2.1)	1 (2.3)	2 (4.5)
**Cerebellar**	1 (1.8)	0 (0.0)	1 (2.6)	1 (2.3)	1 (2.1)	1 (2.3)	1 (2.3)
**NIHSS, admission mean (SD)**	6 (6)	5 (5)	5 (5)	6 (5)	6 (6)	7 (6)	7 (5)
**FMA-UE, at 3 days, mean (SD)**	45 (19)	55 (7)	54 (10)	51 (14)	44 (20)	43 (19)	43 (19)
**FMA-UE sensation, at 3 days, mean (SD)**	10 (4)	12 (1)	11 (3)	11 (3)	10 (4)	10 (4)	10 (4)
**Physical or occupational therapy, n (%)**							
**None**		0	10 (25.6)	15 (34.9)	23 (48.9)	27 (61.4)	31 (70.5)
**1 time / week**		0	0	3 (7.0)	5 (10.6)	4 (9.1)	6 (13.6)
**2 times or more / week**		26 (100)	29 (74.4)	25 (58.1)	19 (40.4)	13 (29.5)	7 (15.9)
**FMA-UE at each time point, median (Q1;Q2)**		56 (51.2;61)	62 (57;64)	64 (61;65.5)	65 (60.5;66)	64 (61;66)	64 (60;66)

*Abbreviations NIHSS*, National Institutes of Health Stroke Scale, *FMA-UE* Fugl Meyer Assessment of Upper Extremity, *Q1*1st quartile, *Q3* 3rd quartile

### Stroke versus healthy controls

Movement time differed significantly from the healthy participants at 3 days and 10 days after stroke but reached almost the same level as the healthy participants, for the remaining timepoints (Table 2). Compared to the healthy controls, the participants with stroke had more movement units and showed a lower peak hand velocity up to 4 weeks after stroke. It could also be observed that the number of movement units was different in stroke compared to healthy controls at 12 months after stroke. The peak elbow angular velocity, arm abduction during drinking and trunk displacement remained different from healthy controls at every time point during the first year posy stroke.

**Table 2:** The median and Quantile–Quantile range of kinematic variables in stroke patients at each tested time point and in healthy controls at one time point

	Movement time, s, median[Q, Q]		Movement units, n, median[Q, Q]		Peak hand velocity, cm/s, median[Q, Q]		Time to peak velocity, %, median[Q, Q]		Peak elbow angular velocity, °/s, median[Q, Q]		Arm abduction, °, median[Q, Q]		Trunk displacement, mm, median[Q, Q]	
**Healthy Controls**	6.4	[5.8, 7.4]	6	[5.7, 7.0]	62.2	[54.6, 67.9]	43.9	[39, 48.1]	103.3	[95.5, 126.2]	29.6	[23.4, 36.1]	28.8	[19.5, 42.9]
**Time post stroke**														
3 days	9.4	[7.7, 11.8]***	10.8	[9.1, 14]***	45.0	[39.6, 55.8]***	37.1	[29.6, 44.2]*	75.2	[62.3, 92.1]***	34.6	[29.3, 41.9]*	42.5	[32.7, 66.1]***
2 weeks	7.2	[6.2, 8.8]**	7.3	[6, 10.5]**	52.7	[44.4, 61.6]***	41.2	[37.2, 45.0]	78.0	[65.3, 95.5]***	39.4	[33.0, 44.5]***	41.6	[28.6, 60.1]***
4 weeks	7.2	[6.0, 8.6]	7.3	[6.2, 10]**	53.6	[45.5, 63.0]**	41.5	[36.2, 45.8]	82.6	[67.2, 94.5]***	35.6	[31.2, 43.3]**	37.7	[26.1, 62.7]**
3 months	6.7	[5.8, 7.7]	6.3	[5.5, 8.5]	57.0	[50.5, 66.1]	42.3	[37.0, 47.3]	87.1	[69.8, 101.9]***	35.9	[30.0, 44.8]**	44.3	[29.7, 56.5]***
6 months	6.9	[5.9, 7.7]	7.0	[5.7, 8.4]	58.4	[48.8, 66.0]	40.1	[34.1, 44.8]	95.0	[77.1, 108.2]**	33.4	[29.7, 40.9]*	38.1	[24.9, 51.7]*
12 months	7.3	[5.8, 7.9]	7.5	[5.7, 10.3]*	55.8	[50.6, 61.9]	41.0	[36.3, 45.1]	89.5	[65.5, 105.6]***	35.8	[29.1, 42.0]*	35.1	[28.5, 67.2]**

[Q, Q] – [Lower quantile, upper quantile]

Statistical differences between healthy controls and stroke patients were tested with Mann-Whitney U test and corrected for multiple comparisons using the method of Holm. Significant differences from healthy controls were found at the early time points for movement time, movement units and peak hand velocity, whereas the peak elbow angular velocity, arm abduction and trunk displacement differed from the healthy controls at every time point

*p < 0.05; **p < 0.01; ***p < 0.001

### Longitudinal changes in stroke

**Fig. 2:**
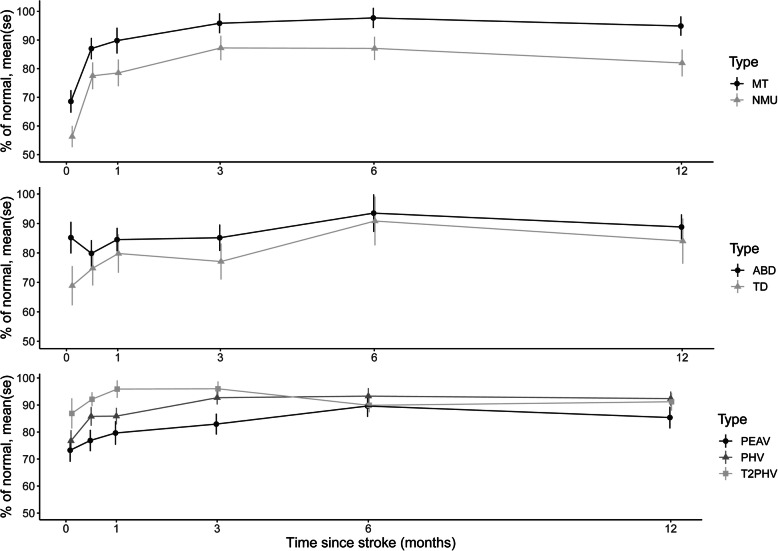
The effect of time on kinematic variables. A value of 100 represents the median result of the healthy participants. Higher values represent better motor function. Abbreviations: ABD, Arm abduction during drinking; MT, Total movement time; NMU, Number of movement units; TD, Trunk displacement; PEAV, peak angular velocity; PHV, Peak Hand Velocity; T2PHV, relative time to Peak Hand Velocity. The variables are shifted 1 point for better visualization

Figure 2 shows the longitudinal changes of the kinematic variables, and Table 3 shows the results of a comparison of these values with the timepoint of best performance using linear mixed models analyses. The best performance for time to peak hand velocity was reached at 3 months after stroke. For the rest of the kinematic variables, the best performance was reached at 6 months after stroke. The movement time, number of movement units, peak elbow angular velocity, peak hand velocity, and trunk displacement showed the largest impairment at 3 days after stroke and remained significantly different from the 6 months reference value (the best value) during the first 4 weeks after stroke (Fig. 2, Table 3). The relative time to peak velocity was significantly different from the 6-month reference point only at 3 days post stroke. No statistically significant change could be detected in the arm abduction. A slight but non-significant decrease could be noted in most of the kinematic variables between 6 and 12 months after stroke. The subgroup analysis of subjects who were included at 4 weeks or later showed a similar trend as in the presented results.

**Table 3:** Results from the linear mixed models analysis showing longitudinal development of arm kinematics over the first year after stroke

	Movement time (S)		Movement units (n)		Peak hand velocity (cm/s)		Time to peak velocity (%)		Peak elbow angular velocity (°/s)		Arm abduction (°)		Trunk displacement (mm)	
	β	***p***-value	β	***p***-value	β	***p***-value	β	***p***-value	β	***p***-value	β	***p***-value	β	***p***-value
(Intercept)	98.5	0.000	86.6	0.000	94.4	0.000	96.7	0.000	90.7	0.000	90.8	0.000	90.8	0.000
Time since stroke														
12 months	−2.1	0.950	−4.6	0.614	0.1	1.000	−4.8	0.524	−3.4	0.756	−1.4	1.000	−6.1	0.830
6 months	(*)		(*)		(*)		−5.7	0.337	(*)		(*)		(*)	
3 months	−2.0	0.955	−0.1	1.000	0.0	1.000	(*)		−5.6	0.259	−6.0	0.569	−13.1	0.127
4 weeks	−11.6	0.000	−12.3	0.004	−9.7	0.000	−2.2	0.969	−14.5	0.000	−3.1	0.969	−16.5	0.041
2 weeks	−18.0	0.000	−17.5	0.000	−12.4	0.000	−5.4	0.448	−21.1	0.000	−10.9	0.094	−28.6	0.000
3 days	−35.5	0.000	−39.1	0.000	−20.4	0.000	−12.0	0.015	−23.5	0.000	−6.9	0.641	−35.4	0.000

The time point for best performance was used as reference and is marked with (*); the kinematic values were inserted into the model as percentages of the median value in the healthy control group

The β-coefficients represent the difference from the best time point under the assumption that missing values are missing at random

*P*-values were adjusted for multiple comparisons with a simultaneous interference procedure

A *p*-value below 0.05 indicates a significant difference from the reference timepoint

## Discussion

This study measured the longitudinal changes in 7 kinematic variables obtained during a drinking task from 3 days to 12 months after stroke. Movement time, number of movement units, and peak hand velocity reached comparable performance levels with the healthy participants at 3 months after stroke, but peak angular velocity, trunk displacement, and arm abduction remained different from the healthy participants during the first year after stroke. Improvements in movement time, number of movement units, peak angular velocity of the elbow, peak hand velocity and trunk displacement occurred predominantly within the first 3 months after stroke. The performance in most variables, however, peaked at 6 months after stroke, and there was a visible but non-significant decline in performance between 6 and 12 months. The arm abduction and relative time to peak velocity did not improve in the same manner as the other 5 variables. No clear improvement over time was noted for arm abduction during drinking, and as mentioned above, it remained different from healthy controls at all time-points. The relative time to peak velocity was only different from its best value at 6 months at 3 days post stroke, but the values were comparable with healthy controls at all timepoints.

The observed improvements in both movement time and movement units (smoothness) are comparable to previously reported longitudinal data in stroke [16, 17]. Van Kordelaar et al. [16] reported improvements in movement time and smoothness during the first 8 weeks after stroke followed by a plateau phase of up to 6 months (26 weeks), with the largest improvements observed during the first 4 weeks after stroke. Similarly, the current study shows a clear improvement in both movement time and number of movement units up to 4 weeks after stroke; the plateau phase was reached at 3 months; and the best performance observed at 6 months after stroke. Interestingly, a common but non-significant trend that showed a slight decline in kinematic measures between 6 and 12 months after stroke was observed. However, the improvements between 3 and 6 months and declines between 6 and 12 months were not statistically significant and were below the clinically meaningful changes of 2.5 s in movement time and 3 movement units as previously reported [18]. Van Dokkum et al. [17] showed gradual improvements in movement time and number of velocity peaks for up to 6 weeks after inclusion in a cohort of patients selected for rehabilitative treatment starting at a mean of 21 days (SD 5 days) after stroke. While the Van Dokkum et al. study [17] focused on patients selected for further treatment, our study followed a non-selected sample of stroke patients admitted to stroke unit care, which strengthens the generalizability of the results. In the present study, we analyzed movement performance during a purposeful functional task including reach, grasp and transport of the glass, and drinking, which is in contrast to previous studies only measuring the reaching [16, 17]. Analyzing performance of the whole task in its natural context, increases the ecological validity of the results. This together with a well-defined study sample adds new knowledge of the behavioral recovery after stroke.

The present study provides longitudinal data for 5 new variables that have not been previously reported. All of these variables are important to capture the overall variation in kinematics after stroke and to discriminate between people with stroke and those without medical conditions affecting their arm functions [14]. The peak hand velocity during reaching showed a similar performance pattern to movement time and number of movement units (smoothness) during the first year after stroke. The similarities between movement time, number of movement units, and peak hand velocity were expected, since all these measures are related to movement velocity. Several studies have shown that strong correlation exists between movement time and smoothness measures [14, 27, 28]. The longer movement time and increased number of movement units, the latter resulting in a less smooth movement, often coexist and are indicators of deficits of in movement control and movement execution. Recruitment of secondary motor areas of the brain have also shown to be associated with less smooth movement during reaching and grasping after stroke [27]. Previous work, similarly to our study, shows that improvements of movement time and smoothness follow the same pattern, which indicates that they both most likely are connected to the neural impairments of the brain accountable to produce a smooth and well-paced reach and grasp movement [16, 27]. More research is, however, needed to determine the impact of these co-existing deficits, movement time and smoothness, on each other.

Healthy subjects show, in general, a smooth bell-shaped velocity profile with one prominent velocity peak during each movement phase [14, 29]. A pre-planned and well-controlled movement production allows for a smooth exponential increase of velocity. If motor impairment is present after stroke it may be difficult to reach an adequate peak velocity. In our study the peak hand velocity was a good indicator of improvement in kinematic performance in the subacute phase, but similarly to movement time and in contrast to number of movement units, the ceiling effect was reached at 3 months and onward after stroke. The relative time to peak hand velocity at 3 days after stroke was the only timepoint that significantly differed from the best value reached at 3 months after stroke. The location of the peak velocity reflects the movement strategy used for reaching an object [29]. An increased precision constraint in task performance results in an earlier change between acceleration and deceleration. Individuals with stroke often show a longer deceleration phase in reaching tasks compared to healthy individuals, which indicates an increased reliance on feedback in the second phase of a reaching task when approaching the object to be grasped [14, 29, 30]. This movement strategy may be associated with stroke-related sensorimotor or visuo-perceptual impairments that are present early after stroke and that resolve over time [31]. Because the relative time to peak velocity did not differ significantly from the healthy participants at any timepoint, it may be less important in the analysis of motor impairment after stroke.

The peak angular velocity of the elbow also improved during the first 3 months after stroke but remained well below the normal values at every timepoint. The peak angular velocity discriminates between healthy individuals and patients with stroke in both acute and chronic stages [14, 19]. From an isokinetic study, we know that stroke patients are less capable of producing extension torque at higher velocities [32]. Extension torque may be diminished after stroke due to reduced agonist activation [33], antagonist co-contraction [34, 35], increased stretch-reflex excitability [34, 36, 37] and/or structural changes within the muscles [38]. The peak angular velocity may be an indicator of reduced force production due to one or more of these impairments. Because the peak angular velocity of the elbow in the stroke patients was well below the healthy participants even in the well-functioning stroke patients with smooth movement, it may reflect remaining impairment after stroke and should be included in kinematic evaluations.

There is an ongoing discussion about importance to enable distinction between motor control elements, muscle strength and compensation when it comes to determination of motor recovery after stroke. Cortes et al. [39] showed that a global composite kinematic metric obtained in 2D antigravity supported reaching, in contrast to clinical assessments, did not show any improvements beyond 5 weeks post stroke. This suggest that most of the improvements in motor function after the first 4 to 5 weeks could only be accounted for improvements in strength and/or or by learning to use new movement strategies. The further and later improvements in strength can be explained be the descending pathways other that cortico-spinal tract [39]. It is also clear that the movement constraints are very different in 2D supported planar reaches and in 3D functional tasks, such as drinking from a glass. The requirements for motor control are also larger in a more complex functional task when the gravity and stability of the body needs to be taken account. In the current study, in most of the kinematic measures the plateau was reached by 3 months post stroke, which is in concurrence with previous research in functional reaching tasks. Although, hypothetically, it is possible that the extra requirements of the 3D functional task (gravity, stability and manipulation of the object) reflect a combination between motor control elements and antigravity strength, which can explain the improvements seen beyond the 4 weeks.

Trunk displacement showed improvements during the first 3 months after stroke. However, the smaller changes observed at later timepoints were not statistically significant and were below the clinically meaningful change of 20 mm reported [18]. A compensatory forward displacement of the trunk is commonly used by individuals with stroke during reaching tasks to improve hand movement performance [40–43]. An excessive use of the trunk to accomplish reaching tasks is common, even in the chronic stage of stroke [12, 44], which is also confirmed in our study. The inclusion of trunk displacement in the 3D kinematic analysis in tasks within the arms workspace in patients with stroke is essential, because trunk movement may interact with other kinematic variables and possibly mask impairments of the shoulder, elbow, or hand function.

Another compensatory movement pattern, the increased use of arm abduction during drinking, remained different from the healthy participants throughout the 12-month follow-up period. Increased shoulder abduction is common after stroke [14, 45–47] and is likely related to an excessive synergetic muscle-activity pattern [48]. Patients with stroke adapt their movement solutions to compensate for the newly acquired motor or sensory deficits. The new movement solutions are learned over a certain period of time and may explain our observation of larger compensatory arm abduction at 10 days after stroke compared to 3 days after stroke. Utilization of compensatory movement patterns over prolonged time after stroke suggest that arm abduction along with trunk displacement should be included as core kinematics when daily reach and manipulation tasks are used in future stroke recovery and intervention trials.

### Strengths and limitations

The kinematic expression of the variables included in this study is highly dependent on the task performed. By examining a natural functional task, we increase the ecological validity of the results, but the results are not necessarily comparable to other results only examining reaching kinematics in a more experimental setup.

The results of the current study are applicable for individuals with moderate to mild stroke (FMA-UE score at least 32 points), who were able to use their more-affected arm to grasp the glass and perform the full drinking task. While most of the patients (73%) entered the study within 10 days post stroke, 18% regained sufficient motor function to perform the drinking task between 4 weeks and 3 months. In addition to those who were able to perform the drinking task within10 days post stroke (73%), we also included patients who within the first 3 months post stroke regained sufficient motor function to perform the drinking task (18%). This later entrance to study might have influenced the results, although the separate subgroup analysis performed for this subgroups showed similar results as seen in the analysis with the entire group. Thus, the results of this study are not only applicable for those who have relatively good motor function already early after stroke, but also for those who show recovery within the first 3 months post stroke. Moreover, even when the study included 6 measurement points during the first year post stroke, the time between the later time points was relatively long, which weakened our ability to verify the exact time when the plateau occurred in our population. A previous study showed that movement time and smoothness plateaued within 8 weeks after stroke [16], and in our study the plateau for these variables was reached somewhere between 4 weeks and 3 months.

## Conclusions

The results from ours study showed that the number of movement units, peak angular elbow velocity, trunk displacement and arm abduction were the most sensitive variables to identify remaining movement deficits during the first year after stroke. The production of movement during a functional task is complex. Kinematic variables are dependent on each other and even if some variables are suitable to describe patients on a group level, other variables may be important for individual recovery. In the individual level, these four kinematic (number of movement units, peak elbow angular velocity, and arm abduction) measures might be appropriate for distinguishing between the true behavioral recovery and compensation.

Researchers and clinicians should take note that some movement performance variables may remain impaired even when the task is completed within sufficient time, and not be satisfied without considering the “hidden” impairments of movement quality. The performance in most of the kinematic variables peaked at 6 months after stroke and then showed a slight decline; this may have been the result of a reduced focus from health care services or reduced attention to motor performance by the patient. Other factors, such as spasticity or fatigue, may be introduced in the later stages of rehabilitation after stroke and complicate motor performance. Clinicians should be aware of the larger specter of motor impairments shown in our study and take these into consideration when evaluating upper-extremity motor performance after stroke.

In conclusion, there is a need for clinically feasible kinematic measures that can enable clinicians to evaluate these impairments. In addition to the established measures, such as movement time and number of movement units, we recommend that the peak angular velocity, arm abduction, and trunk displacement will be included in the analysis of functional tasks in future stroke recovery and intervention trials, as they may capture important variations of motor deficits after stroke.

## Data Availability

Complete data cannot be made publicly available for ethical and legal reasons, according to the Swedish regulations (https://etikprovningsmyndigheten.se/for-forskare/vad-sager-lagen/). Public availability would compromise participant confidentiality or privacy. Data may be released upon request if the purpose is according to the consent given by the participants. Please contact the University of Gothenburg, Sahlgrenska Academy, Institute of Neuroscience and Physiology, Head of Department. [Box 430, 405 30 Göteborg].

## Abbreviations

FMA-UE: Fugl Meyer Assessment of Upper Extremity
FMA-Sensation: Fugl Meyer Assessment domain for sensation, upper extremity
SALGOT: The Stroke Arm Longitudinal Study at the University of Gothenburg
NIHSS: National Institutes of Health Stroke Scale
SD: Standard deviations
Q1: 1st quartile
Q3: 3rd quartile
